# *Addendum*: Li, Y.; Jing, J.; Jin, H.; Qiao, W. Building Keypoint Mappings on Multispectral Images by a Cascade of Classifiers with a Resurrection Mechanism. *Sensors* 2015, *15*, 11769–11786

**DOI:** 10.3390/s151229784

**Published:** 2015-12-02

**Authors:** Yong Li, Jing Jing, Hongbin Jin, Wei Qiao

**Affiliations:** School of Electronic Engineering, Beijing University of Posts and Telecommunications, Rd. Xitucheng 10#, Beijing 100876, China; jingheiieh@bupt.edu.cn (J.J.); jinhongbin@bupt.edu.cn (H.J.); qiaowei@bupt.edu.cn (W.Q.)

The authors wish to update the Acknowledgments in their paper published in *Sensors* [1], doi:10.3390/s150511769, website: http://www.mdpi.com/1424-8220/15/5/11769.
